# An Improved Quantitative Approach for the Assessment of Mitochondrial Fragmentation in Chemoresistant Ovarian Cancer Cells

**DOI:** 10.1371/journal.pone.0074008

**Published:** 2013-09-09

**Authors:** Lee Farrand, Ji Young Kim, Akechai Im-Aram, Jeong-Yong Suh, Hyong Joo Lee, Benjamin K. Tsang

**Affiliations:** 1 World Class University Major in Biomodulation, Department of Agricultural Biotechnology, College of Agriculture and Life Sciences, Seoul National University, Republic of Korea; 2 Department of Obstetrics & Gynaecology, University of Ottawa, Ottawa, Ontario, Canada; 3 Department of Cellular & Molecular Medicine, University of Ottawa, Ottawa, Ontario, Canada; 4 Interdisciplinary School of Health Sciences, University of Ottawa, Ottawa, Ontario, Canada; 5 Chronic Disease Program, Ottawa Hospital Research Institute, Ottawa, Ontario, Canada; Florida International University, United States of America

## Abstract

Mitochondrial fission is a process that involves cleavage of mitochondria into smaller fragments and is regulated by the GTPase Dynamin-related protein 1 (Drp1). Higher levels of mitochondrial fission are associated with the induction of apoptosis in cancer cells. However, current methods to accurately quantify mitochondrial fission in order to compare therapeutics that target this process are often ambiguous or rely on subjective assessment. Mitochondria are also prone to aggregation, making accurate analysis difficult. Here we describe an improved approach for the quantification of mitochondrial fragmentation involving several differences from currently existing methods. Cells are first subjected to cytological centrifugation, which reduces cellular z-axis height and disperses individual mitochondria for easier observation. Three commercially available fluorescence analysis tools are then applied to disambiguate remaining mitochondrial clusters that require further inspection. Finally, cut-off scoring is applied, which can be tailored to individual cell type. The resultant approach allows for the efficient and objective assessment of mitochondrial fragmentation in response to treatment. We applied this technique to an experimental question involving chemosensitive and chemoresistant ovarian cancer (OVCA) cells. Cisplatin and the phytochemical piperlongumine were found to induce both mitochondrial fission and apoptosis in chemosensitive cells, while only piperlongumine was able to elicit these cellular responses in chemoresistant cells. Piperlongumine-induced apoptosis appeared to be mediated by Drp1-dependent mitochondrial fission since the apoptotic response was attenuated by the presence of the Drp1 inhibitor mDivi-1. Our study provides groundwork for a more objective approach to the quantification of mitochondrial fragmentation, and sheds further light on a potential mechanism of action for piperlongumine in the treatment of chemoresistant OVCA.

## Introduction

Mitochondria are dynamic organelles found in most eukaryotic cells that undergo the processes of fission (dividing into separate structures) and fusion (merging of two or more adjacent structures). Fission is known to precede apoptosis in a number of cell types, and is thought to facilitate a more rapid release of mitochondrial pro-apoptotic factors, including cytochrome c and smac [1]. However, the accurate quantification of mitochondrial fission is technically challenging due to the sheer number present in many cell types, and their morphological features. Cells typically exhibit varying degrees of fission depending on the cell type and environmental context [2].

The majority of current approaches for the quantitative assessment of mitochondrial fission are variations of two overarching strategies [2]–[7]. The first approach requires measurement of the lengths of individual mitochondria to determine the degree of fission [3]–[5]. We have found that aggregation of mitochondria [6], [7], especially coiling and knotting of the structures [8], as well as the large number of mitochondrial fragments within each cell renders this approach impractical for at least some cell types. It also fails to take into account the 3-dimensional structure of mitochondria within cells, which does not allow measurement along the z-axis if a single 2-dimensional image is used. Another approach requires the subjective judgment of mitochondrial morphology, and requires the operator to show images that are deemed to be representative of cells with tubular or fragmented mitochondria (other terms used to describe morphology include elongated, fused, intermediate, punctuated and ‘grainy’). Quantification using this approach is essentially based on the opinions of the observer, and each cell is categorized according to which representative image it more closely resembles [9]–[11]. A major concern in applying this technique is its reliance on subjective decision which introduces variability between individual observers. Traditional immunocytochemistry in chambered vessels also produces images that typically contain at least some mitochondria that are out of focus during fluorescence imaging, and therefore incomplete representation of the entire specimen.

The objective of the present study was to develop an improved method to quantify the degree of mitochondrial fragmentation within cells, and to apply this approach in comparing the influence of the phytochemical piperlongumine on mitochondrial fission in chemosensitive and chemoresistant ovarian cancer cells *in vitro*. While the quantification of mitochondrial fragmentation would conceivably account for small mitochondrial fragments appearing due to the synthesis of new mitochondria and the fragmentation caused by mitophagy, when combined with further validation using appropriate markers, more accurate assessments of the extent of mitochondrial fission can be made. Our method involves cytological centrifugation that captures apoptotic and healthy cells using 3-dimensional imaging (via confocal laser microscopy z-stacking), and produces flattened cells with dispersed mitochondria that are easier to distinguish during imaging. In cases of mitochondrial aggregation, three commercially available software tools (Fluorescence Intensity Profiling, Orthogonal Sectioning, and Heat Mapping) are used to disambiguate the clusters and identify numbers of individual mitochondria. In addition, a cut-off scoring technique is applied to more efficiently assess cells as being either fragmented or tubular.

We sought to demonstrate the practical application of our new approach, by using it to investigate the role of mitochondrial fission in chemoresistant ovarian cancer (OVCA). Resistance to CDDP (cisplatin: cis-diamminedichloroplatinum(II)) in ovarian cancer is a significant obstacle to successful treatment [12]. While the involvement of the mitochondria in caspase-mediated apoptosis has been extensively investigated, the role of mitochondrial morphology in chemoresistance remains to be fully understood. Piperlongumine is a natural constituent of the Long Pepper (*Piper longum* L.) and has been reported to exhibit a number of bioactive effects including the inhibition of platelet aggregation [13], downregulation of androgen receptors [14] and broad effects against a range of chemoresistant cancer cell types through the induction of reactive oxygen species [15]. We have applied the above-described quantitative approach to compare the effects of CDDP and piperlongumine on mitochondrial fission and apoptosis in chemosensitive and chemoresistant OVCA cell lines.

## Materials and Methods

### Reagents

CDDP was purchased from Sigma-Aldrich (St Louis, MO, USA). Piperlongumine was purchased from Tocris Bioscience (Bristol, UK). Anti-GAPDH, anti-Drp1 and anti-phospho-Drp1 (Ser637) antibodies were from Cell Signaling Technology (Beverly, CA, USA). Anti-TOM20 antibody was from Santa Cruz Biotechnology (Dallas, TX, USA). Alexa Fluor® 488 secondary antibody, TEMED, RPMI 1640 culture media, fetal bovine serum and ProLong Gold Antifade Reagent with DAPI were from Life Technologies (Carlsbad, CA, USA). All antibodies were diluted in Dako Antibody Diluent (Dako #s0809) from Agilent Technologies (Glostrup, Denmark). Complete Mini Protease inhibitor cocktail tablets and PhosStop phosphatase inhibitor cocktail tablets were obtained from Roche Applied Sciences (Penzberg, Germany).

### Cell Lines and Culture

CDDP-sensitive human OVCA cell line (OV2008) [16] and its resistant counterpart (C13*) [17] were gifts from Drs Rakesh Goel and Barbara Vanderhyden (Ottawa Hospital Cancer Center, Ottawa, ON, Canada), and cultured as previously reported [18]. They are of ovarian endometrioid adenocarcinoma origin with squamous differentiation.

### Annexin V Apoptosis Assay

OVCA cells were stained with FITC-conjugated Annexin V (Bioline, London, UK) and propidium iodide to determine the proportion of the cells that were at the early and late phases of apoptosis [19]. The stained cells were then analyzed using a BD FACSCalibur Flow Cytometer (BD Biosciences) and the CellQuest/ModFit software.

### Immunoblotting and Immunofluorescence Microscopy

Immunoblotting was performed as previously described [18]. Band densities were analyzed and quantified using a BioRad ChemiDoc XRS+ and Image Lab V3.0 (Hercules, CA, USA). OV2008 cells were fixed with 4% paraformaldehyde in 8-well chamber slides, incubated with appropriate fluorescence-conjugated secondary antibodies and stained with ProLong Gold Antifade Reagent with DAPI (blue, nuclear stain). They were imaged immediately with a Zeiss LSM700 confocal scanning microscope equipped with a Zeiss T-PMT digital camera (Zeiss, Oberkochen, Germany).

### Cytological Centrifugation and Fixation of Cells

Cells were seeded for 12 h in RPMI media supplemented with 10% FBS in 6-well plates. Following treatment with appropriate test agents, they were harvested after 2 minutes incubation with 0.025% trypsin (200 µl) at 37°C. The trypsinization was performed quickly to minimize mitochondrial damage and the reaction was stopped with the addition of 10% FBS in RPMI 1640. The cells were gently washed with 1 ml PBS (900×*g*, 1 min; all PBS was filtered with a 0.45 micrometer syringe filter; Sartorius Biotechnology, Goettingen, Germany) and the cell pellet was carefully re-suspended in 500 µl of fresh PBS. Fifty microlitres of the suspension was centrifuged (900×*g*, 4 min) using cytological funnels together with silane-coated glass slides and Whatman filter paper (Hanil Science Cytospin cytological centrifuge; Daejeon, Korea). The cells were then fixed in 4% paraformaldehyde at 4°C for 24 h. They were then washed (3×5 min) in PBS with gentle agitation, permeabilized with 0.02% Triton-X diluted in PBS (incubated for 10 min at 4°C) and subjected to another PBS washing cycle (3×5 min). The cells were incubated with TOM20 (mitochondrial import receptor subunit) antibody (1∶250, 24 h, 4°C), washed with PBS and incubated with Alexa Fluor® 488 secondary antibody (room temperature, 1 h). Cells were then washed in PBS before fixation with a cover slip using ProLong Gold Antifade Reagent with DAPI, and confocal imaging commenced immediately.

### Quantification of Mitochondrial Fission

After cytological centrifugation, fixation and immunostaining of the cells, 3-dimensional images of individual cells were obtained using confocal microscopy by superimposing at least 12 cross-sectional images taken in increments along the Z-axis and encompassing the entire cell volume. Standard exposure settings involved a pixel dwell of >50 microseconds and field dimensions of 300×300 micrometers. A minimum of 100 cells per treatment group were assessed for mitochondrial fission in each replicate, with statistics derived from three independent replicates. Each individual cell analyzed was classified as either fragmented or not-fragmented in accordance with whether it met the cut-off score of 8. Cells that fulfilled the definition of ‘fragmented’ therefore contained 8 or more individual mitochondrial fragments that were each <3 micrometers in length across the longest axis. In cases of ambiguity due to aggregation of the mitochondria, three commercially available software tools included in the Zeiss LSM software package (Fluorescence Intensity Profiling, Orthogonal Sectioning and Heat Mapping) were used to resolve the uncertainties. Fluorescence Intensity Profiling enables the detection of fluorescently labeled mitochondrial boundaries (labeled with TOM20), as reflected by sharp increases or decreases in fluorescence intensity [20]. The operator draws a straight line through any region of the image, and an automated graph of the fluorescence intensity is constructed. Orthogonal Sectioning allows any region in the image to be inspected from an x- or y-axis point-of-view (POV). This creates a virtual cross-sectional perspective and provides information on mitochondrial boundaries that would normally be obscured if viewing from the traditional top-down POV. Heat Mapping color-codes all fluorescent objects within a 3-dimensional image according to the position of the object along the z-axis. In visualizing mitochondria, the tool is most useful for distinguishing separate mitochondria that are located on opposing sides of the nucleus (and would otherwise appear as singular mitochondria). In the majority of cases, these tools were not needed to recognize fragmented mitochondria, as cytological centrifugation dispersed the organelles for easier viewing. The mitochondrial morphology of at least 80 cells per treatment group was determined, with the observer blinded to the identity of treatment groups. Quantifications were derived from three independent experiments.

### Statistical Analysis

Results are expressed as the mean ± SEM of at least three independent experiments. Statistical analysis was carried out by one-way, two-way or three-way analysis of variance, using SigmaPlot software (Versions 12; Systat Software, Chicago, IL, USA). Differences between multiple experimental groups were determined by the Bonferroni post-hoc test. Statistical significance was inferred at *p*<0.05.

## Results

### Cytological Centrifugation Enhances Mitochondrial Imaging by Increasing the Separating Distances between Individual Mitochondria

**Figure 1:**
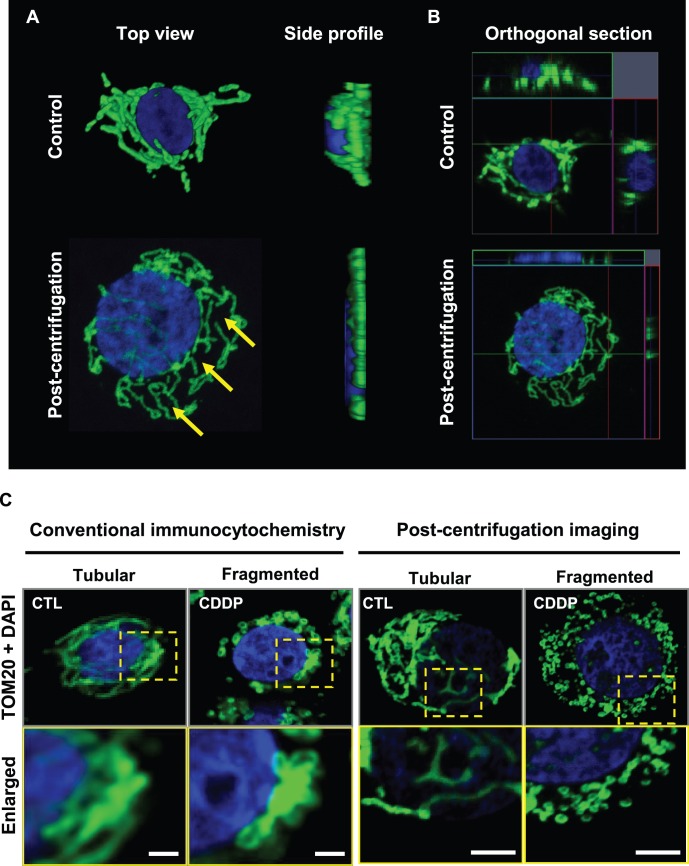
Effect of cytological centrifugation on mitochondrial imaging with confocal microscopy. (**A**) 3-dimensional top view and side profiles of untreated OV2008 cells, with and without cytological centrifugation prior to fixation. Yellow arrows indicate typical areas of separation that appear between mitochondria after centrifugation. (**B**) Orthogonal sections of the same images seen in (A) taken with identical camera exposure settings. Cells were fixed in 3.7% paraformaldehyde and stained for nuclear (DAPI, blue) and mitochondrial (TOM20, green) signal. (**C**) Comparison of mitochondrial image quality between conventional immunocytochemistry and cytological centrifugation approaches. Images of classical tubular and fragmented mitochondrial morphology becomes visibly sharper after centrifugation due to the minimization of fluorescence originating from background sources beyond the focal plane. The enlargement of the nucleus post-centrifugation is due to flattening of the cells by centripetal force. Scale bars = 5 µm.

We first compared the effects of cytological centrifugation on confocal imaging quality of mitochondria. Optimization revealed that the ideal rotor settings of the cytological centrifuge for imaging OV2008 cells included a 900×g spin for 4 minutes. All subsequent experiments were conducted using these settings. In the absence of the centrifugation step, mitochondria were observed to aggregate closely to the nucleus and to curl towards the angle of the viewing lens (Figure 1, Figure S1A). Side profiling of cells without centrifugation revealed a large z-axis height of at least 8 micrometers, making individual distinction of separate mitochondria difficult. With the inclusion of a centrifugation step, the mitochondria were separated further from the nucleus, and increased distances emerged between individual mitochondria. Centrifuged cells exhibited a reduced z-axis height of approximately 3 micrometers, resulting in an easier observation of individual mitochondrial structures (Figure 1B, Figures S1B–C). A comparison of image quality with and without centrifugation, using identical imaging settings, revealed that centrifugation improved the overall clarity of mitochondrial features (Figure 1C). This was due, in part, to the fact that without centrifugation, the fluorescent images included a higher degree of ‘out-of-focus’ background fluorescence (ie. from mitochondria that were not clearly visible within the z-axis plane at which the image was taken). Centrifugation improves image focus by flattening cell features within the same focal plane.

### Fluorescence Intensity Profiling, Orthogonal Sectioning and Heat Mapping Tools Allow for Rapid Distinction of Individual Mitochondrial Fragments within Aggregates

**Figure 2:**
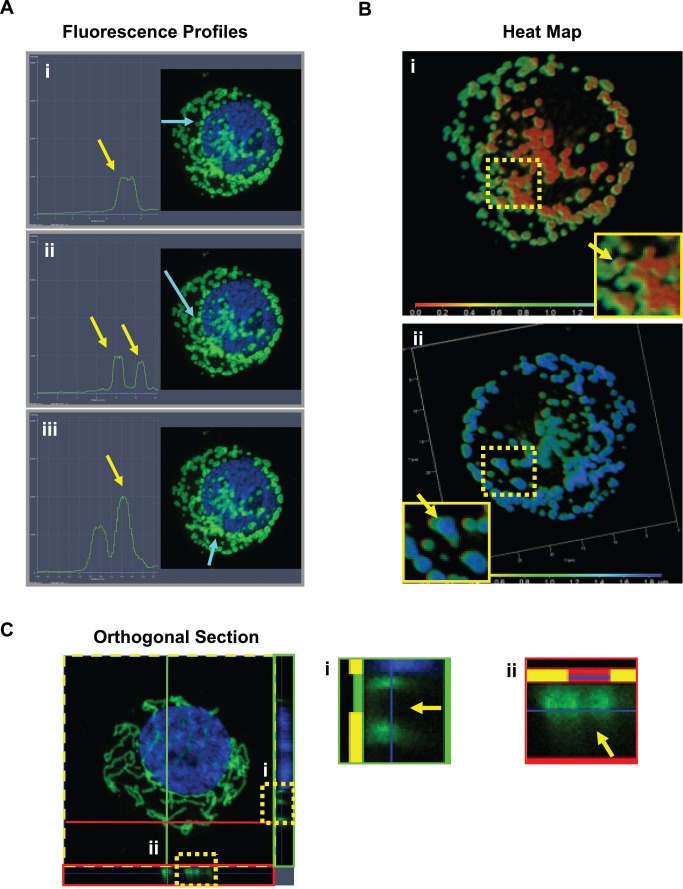
Disambiguation of mitochondrial aggregates using commercially-available fluorescence analysis software tools. (**A**) Fluorescence intensity profiling of a single cell with fragmented mitochondria. Intensity of mitochondrial signal along a linear profile selected by the operator (light blue arrow) is represented quantitatively. (i) Emission peak of a single mitochondrion is indicated graphically (yellow arrow). (ii) Separated mitochondria are evident as independent peaks. (iii) Higher peaks (yellow arrow) suggest the presence of multiple mitochondria, superimposed during centrifugation. 3D image data is layered transparently, with individual mitochondrial number being directly proportional to the intensity of fluorescence. (**B**) Heat mapping of a single cell treated with CDDP (10 µM, 12 h). Differences in z-axis location values of mitochondrial fragments are represented as colors. (i) Magnification (yellow box) revealing distinct mitochondria at different z-axis heights (green vs. orange). (ii) The reverse view of the same cell in (i), indicating further individual fragments (cyan vs blue, yellow arrow). (**C**) Orthogonal section tool showing cross sections of a single cell along the y- axis (inset dotted yellow box, magnified as green box) and x-axis (magnified as red box). Y. (i) Magnification (green box) of y-axis orthogonal section. (ii) Magnification (red box) of x-axis orthogonal section. Yellow arrows indicate spaces separating individual mitochondria.

We noted that although the centrifugation step enhanced our ability to distinguish mitochondria, in some cases aggregation of the mitochondria appeared unavoidable despite efforts to resolve the issue. We adopted three fluorescence analysis tools (Fluorescence Intensity Profiling, Orthogonal Sectioning and Heat Mapping) to supplement the quantification of mitochondria present within aggregates. These tools provide greater information on the spatial orientation of mitochondrial membranes, allowing disambiguation of fluorescent signal that may otherwise seem confusing. Fluorescence Intensity Profiling (Zeiss LSM software package) is a tool normally used to determine signal-to-background ratios across fluorescent regions of an image [20]. This tool can be alternatively used to determine the number of individual mitochondria within an aggregate, as the intensity of fluorescence is directly proportional to the number of mitochondrial membranes. Using transparent overlays, the number of mitochondria can be extrapolated in large groups by simple division of the peak signal by the intensity of fluorescence obtained from a single mitochondrion (Figure 2A). Heat Mapping provides information on z-axis mitochondrial locations using a color-coded spectrum (Figure 2B, Figures S2A–C) [21]. Mitochondria that are superimposed or flattened behind or in front of the nucleus can be easily identified according to their color assignment. Finally, Orthogonal Sectioning (Zeiss LSM) provides cross-sectional views of any x-y location from a perspective perpendicular to the z-axis, allowing close inspection of mitochondrial boundaries that would otherwise be obscured from a traditional top-down view (Figure 2C). At sufficiently high resolution, and with optimal expansion of the cytoplasm via the centrifugation step, we found that these fluorescence tools were only required in >10% of cases, where a decision could not be reached due to irregular mitochondrial aggregation. The effective use of these tools are dependent on several assumptions, for instance, that all mitochondria in an aggregate are equally stained, that fluorescent signal has a sufficient signal to noise ratio and that the mitochondrial membranes are distinguishable by corresponding changes in fluorescent signal.

### Application of Cut-off Scores Allows Distinction between Cells Containing Different Degrees of Mitochondrial Fission

**Figure 3:**
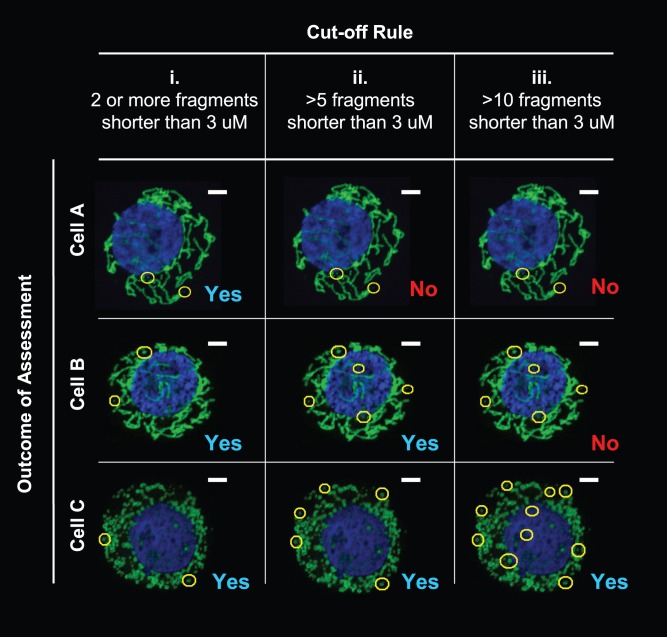
Selection of cut-off scores determines stringency of categorization. Figure shows three cells with varying degrees of fission. (**i**) A cut-off score of 2 (outcome is ‘Yes’, if 2 or more mitochondrial fragments are detected that are <3 micrometers in length across the longest axis) puts all 3 cell types in the same category. (**ii**) A cut-off score raised to 5 distinguishes Cell A as different to Cells B and C (a criterion for classifying Cell A as ‘tubular’) (**c**) A cut-off score of 10 places Cells A and B in the same category, distinct from Cell C. Use of more than one cut-off score allows for the distinction of varying degrees of mitochondrial fission.

Although it is possible to precisely determine the number of mitochondria in any given cell, it is time-consuming and impractical if assessing a large number of cells. We next devised a semi-quantitative approach for a more efficient and less labor-intensive assessment of the images. We proceeded by assessing the mitochondrial phenotype (tubular or fragmented) based on a cut-off score defined by a minimum number of mitochondrial fragments of a defined length present in each cell. The stringency of categorization is determined by the cut-off score chosen (Figure 3). If 8 or more mitochondria that were each shorter than 3 micrometers in length were found anywhere in the cell, the entire cell would qualify as “fragmented”. Fulfillment of the criteria renders further assessment of the remaining mitochondria unnecessary (shown in Figure 3).

### Chemosensitivity to CDDP and Piperlongumine is Associated with Mitochondrial Fission

**Figure 4:**
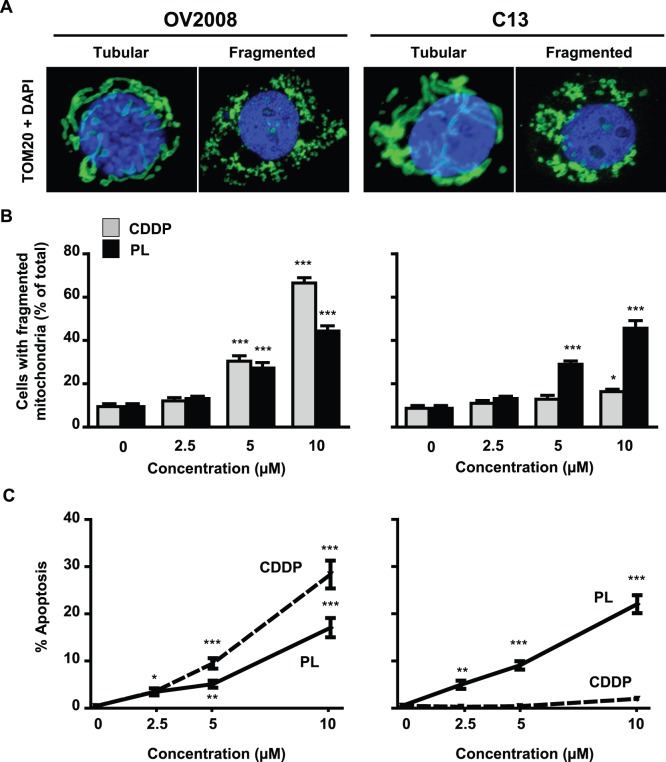
Effects of piperlongumine and CDDP on mitochondrial fission and apoptosis in OVCA cells. (**A**) Representative images of tubular and fragmented mitochondria in chemosensitive cells (OV2008) and their resistant counterparts (C13). Mitochondria and nuclei were stained with TOM20 (green) and DAPI (blue), respectively. (**B**) Effect of piperlongumine and CDDP on mitochondrial fission in OV2008 and C13 cells, as assessed with a single cut-off score of 8 (cells containing 8 or more mitochondrial fragments <3 micrometers in length were classified as having fragmented mitochondria). (**C**) Effect of CDDP and piperlongumine on apoptosis, as assessed by Annexin V assay (*, *p*<0.05; **, *p*<0.01; ***, *p*<0.001 versus respective untreated control).

We next applied this technique to investigate the influence of piperlongumine and CDDP (0 and 10 µM, 12 h) on mitochondrial fission in chemosensitive (OV2008) and chemoresistant (C13) OVCA cells (Figure 4). Both compounds caused increases in mitochondrial fission and apoptosis in a concentration-dependent manner in OV2008. However, only piperlongumine had the same effect in C13, suggesting that the latter not only promotes apoptosis but also induces mitochondrial fission in chemoresistant OVCA cells. A minimum of 100 cells per treatment group were assessed for mitochondrial fission in each replicate, with statistics derived from three independent replicates.

### CDDP and Piperlongumine-induced Mitochondrial Fission and Apoptosis are Drp1-dependent in Chemosensitive OVCA

**Figure 5:**
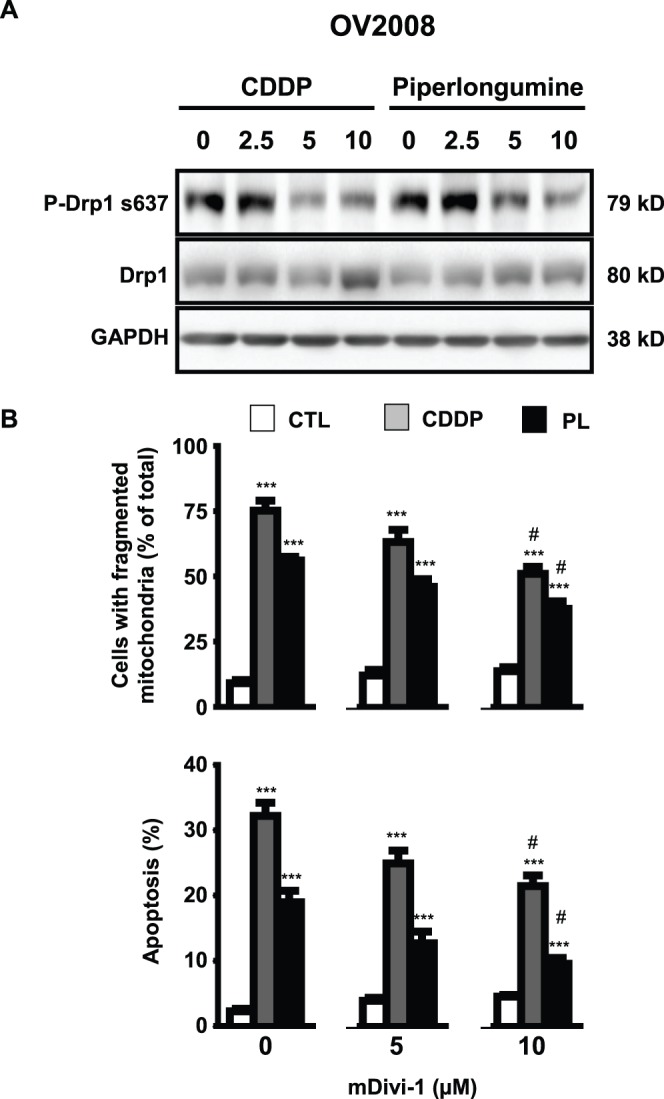
CDDP- and piperlongumine-induced mitochondrial fission and apoptosis is Drp1-dependent in chemosensitive OVCA cells. (**A**) CDDP (10 µM) and piperlongumine (10 µM) down-regulated of phospho-Drp1 (Ser637) content in OV2008 cells *in vitro*. (**B**) (**i**) Effect of mDivi-1 (0–10 µM) on CDDP- and piperlongumine-induced mitochondrial fission and (**ii**) apoptosis as assessed by Annexin V assay (**, *p*<0.01; ***, *p*<0.001 versus respective DMSO control treated with identical mDivi-1 concentration, #, *p*<0.05 versus respective PL or CDDP treatment in the absence of mDivi-1).

In response to various types of cell stress, Drp1 is activated by dephosphorylation at Ser637. This is followed by its recruitment to the mitochondria where it oligomerizes to provide the mechanical strength needed for fission to occur [22]. We hypothesized that if Drp1-dependent mitochondrial fission is a determinant of the chemosensitive response, both CDDP and piperlongumine treatment should result in dephosphorylation of Drp1. This was indeed the case in OV2008 cells (Figure 5A). More importantly, treatment of OV2008 cells with a pharmacological inhibitor of Drp1, mDivi-1, significantly attenuated both mitochondrial fission and apoptosis induced by the two compounds (Figure 5B). This data supports a causative link between fission and apoptosis. A minimum of 100 cells per treatment group were assessed for mitochondrial fission in each replicate, with statistics derived from three independent replicates.

### Drp1-dependent Mitochondrial Fission is a Determinant of Piperlongumine-induced Apoptosis in Chemoresistant OVCA

**Figure 6:**
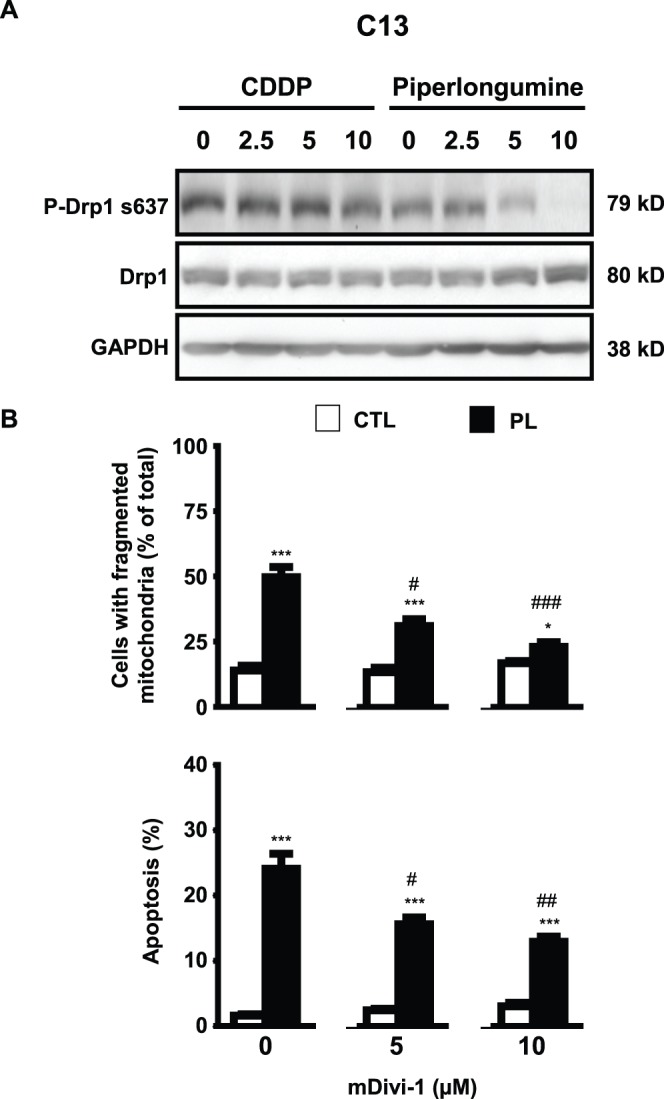
Piperlongumine induces Drp1-dependent mitochondrial fission and apoptosis in CDDP-resistant ovarian cancer cells. (**A**) Piperlongumine but not CDDP (0–10 µM) down-regulates phospho-Drp1 (Ser637) content. (**B**) (**i**) Effect of mDivi-1 (0–10 µM) on piperlongumine-induced mitochondrial fission and (**ii**) apoptosis, as assessed by Annexin V assay (**, p<0.01; ***, p<0.001 versus respective DMSO control treated with identical mDivi-1 concentration, #, *p*<0.05; ##, *p*<0.01; ###, *p*<0.001 versus respective PL or CDDP treatment in the absence of mDivi-1).).

Our earlier studies have shown that mitochondrial fission is associated with chemosensitivity, while CDDP resistance is marked by a lack of such activity (Figure 4). We next sought to determine whether piperlongumine was causing apoptosis in chemoresistant C13* cells via Drp1-dependent mitochondrial fission. Concentration-response analysis showed that piperlongumine, but not CDDP, induced dephosphorylation of Drp1 at Ser637 (Figure 6A). Addition of the Drp1-inhibitor mDivi-1 also partially attenuated piperlongumine-induced mitochondrial fission and apoptosis (Figure 6B). A minimum of 100 cells per treatment group were assessed for mitochondrial fission in each replicate, with statistics derived from three independent replicates.

## Discussion

While the quantification of mitochondrial fission has been undertaken in several well-designed studies, the descriptions of published approaches are somewhat ambiguous and subjective [2]–[7]. We note two significant difficulties in accurately quantifying fission. The first is aggregation of the mitochondria, particularly around the nucleus, which makes distinction of separate organelles difficult. The second is the time taken to quantify the mitochondria, even if all individual fragments can be distinguished using advanced 3-dimensional mapping techniques.

We acknowledge the existence of several published automated methods which we have investigated and found to be interesting. However, we believe that these methods require extensive background knowledge on computational algorithms in order to be used effectively. One method uses automated fluorescent pulsing at 30 Hz and the employment of computer-aided analysis [23]. In our attempt to follow the steps provided by the researchers, we found considerable difficulty in interpreting a number of the instructions which require considerable background knowledge, and were unable to succeed in producing meaningful data. The generation of a synthetic binary image as a test model also requires extensive familiarity with appropriate dimensions of the mitochondria in each cell type, a requirement not needed for our approach.

Another intriguing computational approach [24] involves automated subtyping of mitochondrial morphology. We have also attempted to employ the MicroP software written by the authors for our experimental needs, however, it was similarly very difficult for us to learn due to the technical (computational knowledge) needed. MicroP requires the user to successfully calibrate the software before use.

**Figure 7:**
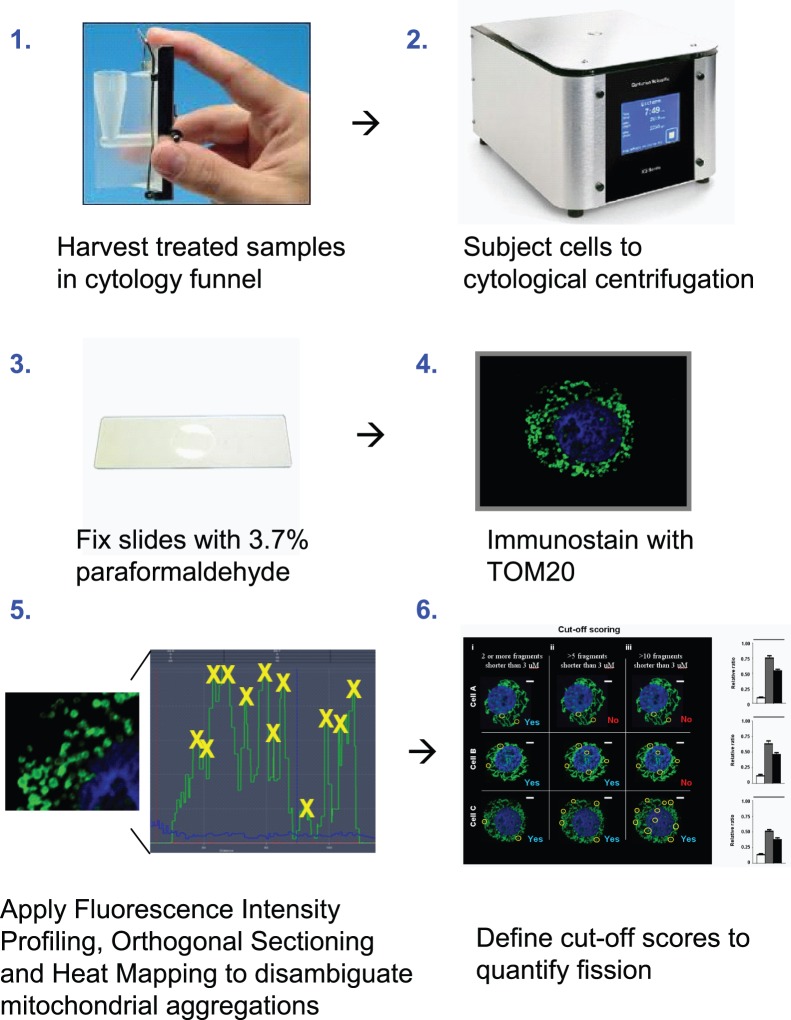
Overview of the novel approach, involving cytological centrifugation, fluorescence analysis and assignment of cut-off scores for mitochondrial fission quantification.

In the present studies, we have addressed the issue of aggregation by applying a cytological centrifugation step, which flattens the cytoplasm along the plane of the slide and renders distinction of individual mitochondria significantly easier. The issue of time constraint was addressed by the application of cut-off scoring to allow for less labor-intensive categorization of mitochondrial phenotypes. While the most accurate method for quantification of fission would be an absolute count of the total number of individual fragments, the time required makes this approach impractical, especially for studies with a large sample size. Our suggested approach allows for the acquisition of mitochondrial fission data comparable to that required in prior study designs [2]–[7], but with more specific guidelines for independent reproducibility. An overview of the new approach is shown in Figure 7.

While our approach offers obvious advantages, we also note several potential pitfalls. The diversity in mitochondrial morphology across cell types is significant and may require fine adjustment and validation of the protocol. For example, some cell types may have a large number of mitochondria and a relatively lower volume of cytoplasm. In such cases, careful optimization of the centrifuge step and a larger number of z-axis images per cell may be needed. Higher centrifuge speeds enhance mitochondrial dispersion, but can also promote rupture of the nucleus (shown in Figure S3). We have also attempted to use our approach with *in vivo* OVCA tumor samples stained with TOM20, and found that the inability to centrifuge solid tumors combined with excessive non-specific background signal made it infeasible. A rapid cell harvest prior to centrifugation could also be essential, and we suspect that mitochondria may fragment very quickly after harvest. This has implications for the number of different samples within a treatment group that can be simultaneously assessed. A possible solution is to request the aid of more operators to assist in the harvest step, to allow for faster completion. It is possible that this approach does not allow for the distinction of mitochondrial fission as a causative factor for the appearance of mitochondrial fragments. Other processes, including the synthesis of new mitochondria and mitophagy could also lead to such events. Therefore, it is important to also employ other approaches related to mitochondrial fission in order to form conclusions. For example, the analysis of mitochondrial fission machinery (such as Drp1 activation) will lend further weight to a hypothesis supported by fission quantification data.

While mitochondrial fission has been linked with apoptotic capacity [25], its relationship with CDDP resistance has not been carefully examined. We found that CDDP and piperlongumine activate Drp1 (via removal of phosphorylation at Ser637) and induce both fission and apoptosis in chemosensitive cells (OV2008). In contrast, only piperlongumine was effective in its resistant counterparts (C13*). Our observation that the specific Drp1 inhibitor mDivi-1 attenuated both piperlongumine-induced fission and apoptosis provided further support for hypothesis that CDDP resistance may in part be a consequence of Drp1 inactivation and the resultant suppression of mitochondrial fission. Further studies are required to better understand how the dysregulation of mitochondrial fission plays a role in CDDP resistance, and to provide insights for new therapeutic strategies to overcome such resistance in OVCA.

In summary, we have developed and tested a novel approach for the quantification of mitochondrial fission that we believe is scientifically more rigorous than the methods currently described in the literature. Its advantages include greater accuracy, minimal subjectivity and higher efficiency. In addition, we have demonstrated that piperlongumine induces apoptosis in CDDP-resistant OVCA, and that this phenomenon is associated with the induction of mitochondrial fission. Successful application of this technique relies upon minimal damage to mitochondria during harvest and fixation, cytological centrifugation and trypsinization, as well as the proper application of fluorescence analysis tools. These programs can be purchased online and training materials are also publicly available.

## Supporting Information

Figure S1 **Effect of cytological centrifugation on cell dimensions.** (**A**) Typical OV2008 cells treated with and without CDDP (10 µM), in the absence of a centrifugation step. Aggregation of mitochondria can make quantification challenging. (**B**) OV2008 cell treated with CDDP (10 µM) and subjected to centrifugation (900×*g*, 4 min), causing flattening of the cell without membrane rupture. (**C**) Comparison between cells with and without centrifugation step.
(EPS)

Figure S2 **Advanced aspects of the heat mapping technique.** (**A**) In fixed-axis mode, intensity of signal can be manipulated along a single plane, showing relative z-axis positions of individual mitochondria. Higher intensities indicate greater z-axis height and higher numbers of mitochondria (yellow arrows). (**B**) In rotational mode, color indicates the position of the nucleus, even in the absence of DAPI stain. (**C**) CDDP (10 µM) -treated OV2008 cell showing intensity peaks indicative of mitochondrial aggregations (yellow arrows).
(EPS)

Figure S3 **Effects of centrifuge speed on mitochondrial dispersion and rupture of the nuclear envelope.** Lower speeds (300×g) result in suboptimal dispersion, while high speeds (1200×g) rupture the nucleus (indicated by yellow arrow). Mitochondria and nuclei were stained with TOM20 (green) and DAPI (blue).
(EPS)

Figure S4 **High resolution field scans showing mitochondrial morphology of populations of OV2008 cells.** Single-cell images were taken for the majority of the results purely due to practical considerations. For high resolution laser microscopy imaging, the time taken to capture an image is dependent upon pixel dwell and the resolution chosen. Larger fields of view require a much greater amount of time to capture, thus necessitating our approach of imaging single cell images to gather an adequate amount of data within a reasonable timeframe.(A) Uncentrifuged OV2008 control cells. Not all mitochondria are visible, as the cells were not centrifuged, and a single z-axis scan was captured. Time taken to capture image = approx. 40 minutes. (B) Uncentrifuged OV2008 cells treated with 10 uM CDDP. Not all mitochondria are visible, as the cells were not centrifuged, and a single z-axis scan was captured. Time taken to capture image = approx. 30 minutes. (C) Centrifuged OV2008 control cells. This image was taken prior to completion of our optimization of the approach. Time taken to capture image = approx. 20 minutes. (D) Centrifuged OV2008 cells treated with 10 uM CDDP. This image was taken prior to completion of our optimization of the approach. Time taken to capture image = approx. 10 minutes.
(EPS)

## References

[1] Youle RJ, Karbowski M (2005) Mitochondrial fission in apoptosis. Nature Reviews Molecular Cell Biology 6: 657–663 (doi:10.1038/nrm1697). 16025099 10.1038/nrm1697

[2] Detmer SA, Chan DC (2007) Functions and dysfunctions of mitochondrial dynamics. Nature Reviews Molecular Cell Biology 8: 870–879 (doi:10.1038/nrm2275). 17928812 10.1038/nrm2275

[3] Wang X, Su B, Siedlak SL, Moreira PI, Fujioka H, et al (2008) Amyloid-β overproduction causes abnormal mitochondrial dynamics via differential modulation of mitochondrial fission/fusion proteins. Proceedings of the National Academy of Sciences 105: 19318–19323 (doi:10.1073/pnas.0804871105). 10.1073/pnas.0804871105 PMC2614759 19050078

[4] Barsoum MJ, Yuan H, Gerencser AA, Liot G, Kushnareva Y, et al (2006) Nitric oxide-induced mitochondrial fission is regulated by dynamin-related GTPases in neurons. The EMBO Journal 25: 3900–3911 (doi:10.1038/sj.emboj.7601253). 16874299 10.1038/sj.emboj.7601253 PMC1553198

[5] Yu T, Fox RJ, Burwell LS, Yoon Y (2005) Regulation of mitochondrial fission and apoptosis by the mitochondrial outer membrane protein hFis1. Journal of Cell Science 118: 4141–4151 (doi:10.1242/jcs.02537). 16118244 10.1242/jcs.02537

[6] Sheridan C, Delivani P, Cullen SP, Martin SJ (2008) Bax- or Bak-Induced Mitochondrial Fission Can Be Uncoupled from Cytochrome c Release. Molecular Cell 31: 570–585 (doi:10.1016/j.molcel.2008.08.002). 18722181 10.1016/j.molcel.2008.08.002

[7] Yang Y, Ouyang Y, Yang L, Beal MF, McQuibban A, et al (2008) Pink1 regulates mitochondrial dynamics through interaction with the fission/fusion machinery. Proceedings of the National Academy of Sciences 105: 7070–7075 (doi:10.1073/pnas.0711845105). 10.1073/pnas.0711845105 PMC2383971 18443288

[8] Kuznetsov AV, Hermann M, Troppmair J, Margreiter R, Hengster P (2010) Complex patterns of mitochondrial dynamics in human pancreatic cells revealed by fluorescent confocal imaging. Journal of Cellular and Molecular Medicine 14: 417–425 (doi:10.1111/j.1582-4934.2009.00750.x). 19382913 10.1111/j.1582-4934.2009.00750.x PMC3837585

[9] Yu T, Robotham JL, Yoon Y (2006) Increased production of reactive oxygen species in hyperglycemic conditions requires dynamic change of mitochondrial morphology. Proceedings of the National Academy of Sciences of the United States of America 103: 2653–2658 (doi:10.1073/pnas.0511154103). 16477035 10.1073/pnas.0511154103 PMC1413838

[10] Delivani P, Adrain C, Taylor RC, Duriez PJ, Martin SJ (2006) Role for CED-9 and Egl-1 as Regulators of Mitochondrial Fission and Fusion Dynamics. Molecular Cell 21: 761–773 (doi:10.1016/j.molcel.2006.01.034). 16543146 10.1016/j.molcel.2006.01.034

[11] Ong SB, Subrayan S, Lim SY, Yellon DM, Davidson SM, et al (2010) Inhibiting Mitochondrial Fission Protects the Heart Against Ischemia/Reperfusion Injury. Circulation 121: 2012–2022 (doi:10.1161/CIRCULATIONAHA.109.906610). 20421521 10.1161/CIRCULATIONAHA.109.906610

[12] Akiyama SI, Chen ZS, Sumizawa T, Furukawa T (1999) Resistance to cisplatin. Anti-Cancer Drug Design 14: 143–151. 10405641

[13] Iwashita M, Oka N, Ohkubo S, Saito M, Nakahata N (2007) Piperlongumine, a constituent of Piper longum L., inhibits rabbit platelet aggregation as a thromboxane A2 receptor antagonist. European Journal of Pharmacology 570: 38–42 (doi:10.1016/j.ejphar.2007.05.073). 17618620 10.1016/j.ejphar.2007.05.073

[14] Golovine KV, Makhov PB, Teper E, Kutikov A, Canter D, et al (2013) Piperlongumine induces rapid depletion of the androgen receptor in human prostate cancer cells. The Prostate 73: 23–30 (doi:10.1002/pros.22535). 22592999 10.1002/pros.22535 PMC3491117

[15] Raj L, Ide T, Gurkar AU, Foley M, Schenone M, et al (2011) Selective killing of cancer cells by a small molecule targeting the stress response to ROS. Nature 475: 231–234 (doi:10.1038/nature10167). 21753854 10.1038/nature10167 PMC3316487

[16] DiSaia PJ, Sinkovics JG, Rutledge FN, Smith JP (1972) Cell-mediated immunity to human malignant cells. A brief review and further studies with two gynecologic tumors. American Journal of Obstetrics and Gynecology 114: 979–989. 4118903 10.1016/0002-9378(72)90109-3

[17] Andrews PA, Murphy MP, Howell SB (1985) Differential Potentiation of Alkylating and Platinating Agent Cytotoxicity in Human Ovarian Carcinoma Cells by Glutathione Depletion. Cancer Research 45: 6250–6253. 4063975

[18] Asselin E, Mills GB, Tsang BK (2001) XIAP Regulates Akt Activity and Caspase-3-dependent Cleavage during Cisplatin-induced Apoptosis in Human Ovarian Epithelial Cancer Cells. Cancer Research 61: 1862–1868. 11280739

[19] Vermes I, Haanen C, Steffens-Nakken H, Reutellingsperger C (1995) A novel assay for apoptosis flow cytometric detection of phosphatidylserine expression on early apoptotic cells using fluorescein labelled Annexin V. Journal of Immunological Methods. 184: 39–51 (doi:10.1016/0022-1759(95)00072-I). 10.1016/0022-1759(95)00072-i 7622868

[20] Kask P, Palo K, Ullmann D, Gall K (1999) Fluorescence-intensity distribution analysis and its application in biomolecular detection technology. Proceedings of the National Academy of Sciences 96: 13756–13761 (doi:10.1073/pnas.96.24.13756). 10.1073/pnas.96.24.13756 PMC24137 10570145

[21] Pepperkok R, Ellenberg J (2006) High-throughput fluorescence microscopy for systems biology. Nature Reviews Molecular Cell Biology 7: 690–696 (doi:10.1038/nrm1979). 16850035 10.1038/nrm1979

[22] Cribbs JT, Strack S (2007) Reversible phosphorylation of Drp1 by cyclic AMP-dependent protein kinase and calcineurin regulates mitochondrial fission and cell death. EMBO Reports 8: 939–944 (doi:10.1038/sj.embor.7401062). 17721437 10.1038/sj.embor.7401062 PMC2002551

[23] Koopman WJH, Visch HJ, Smeitink JA, Willems PH (2006) Simultaneous quantitative measurement and automated analysis of mitochondrial morphology, mass, potential, and motility in living human skin fibroblasts. Cytometry Part A 69: A1–12 (doi:10.1002/cyto.a.20198). 10.1002/cyto.a.20198 16342116

[24] Peng JY, Lin CC, Chen YJ, Kao LS, Liu YC, et al (2011) Automatic Morphological Subtyping Reveals New Roles of Caspases in Mitochondrial Dynamics. PLoS Comput Biol 7: e1002212 (doi:10.1371/journal.pcbi.1002212). 21998575 10.1371/journal.pcbi.1002212 PMC3188504

[25] Perfettini JL, Roumier T, Kroemer G (2005) Mitochondrial fusion and fission in the control of apoptosis. Trends in Cell Biology 15: 179–183 (doi:10.1016/j.tcb.2005.02.005). 15817372 10.1016/j.tcb.2005.02.005

